# Supplementary material for: MicroRNA Dysregulation in Early Breast Cancer Diagnosis: A Systematic Review and Meta-Analysis
Source: Int J Mol Sci. 2023 May 5;24(9):8270. doi: 10.3390/ijms24098270 (PMC10179484; doi:10.3390/ijms24098270)
Supplement: Supplementary file 1 [file ijms-24-08270-s001.zip › ijms-2272292-supplementary.pdf]

International Journal of
Molecular Sciences

MDPI

Supplementary Materials

MicroRNA Dysregulation in Early Breast Cancer Diagnosis: A Systematic Review and Meta-Analysis

Alejandro Garrido-Palacios, Iñigo María Perez-Castillo, Ana María Núñez-Negrillo, Jonathan Cortés-Martín, Juan Carlos Sánchez-García and María José Aguilar-Cordero

Supplementary Table S1. Evaluation of risk of bias Newcastle-Otawa Scale (case-control design).

Reference	Design	Selection	Comparability	Exposure	Total
[15]	Case-Control	★★★	★★	★★★	8/9
[16]	Case-Control	★★★★	★★	★★★	9/9
[17]	Case-Control	★★★★	★★	★★★	9/9
[18]	Case-Control	★★★	★★	★★★	8/9
[19]	Case-Control	★	★	★★★	5/9
[20]	Case-Control	★★★★	★★	★★★	9/9
[21]	Case-Control	★★★	★	★★★	7/9
[22]	Case-Control	★★★	★★	★★★	8/9
[23]	Case-Control	★★★★	★★	★★★	9/9
[24]	Case-Control	★★★	★★	★★	7/9
[25]	Case-Control	★★★★	★	★★★	8/9
[26]	Case-Control	★★★★	★★	★★★	9/9
[27]	Case-Control	★★★★	★	★★★	8/9
[28]	Case-Control	★★★	★★	★★★	7/9
[29]	Case-Control	★★★★	★★	★★★	9/9
[49]	Case-Control	★★★★	★★	★★★	9/9

[94]	Case-Control	★★★★	★★	★★★	9/9
[30]	Case-Control	★★★★	★★	★★★	9/9
[45]	Case-Control	★★★★	★	★★★	8/9
[31]	Case-Control	★★★★	★★	★★★	9/9
[32]	Case-Control	★★★	★	★★★	7/9
[33]	Case-Control	★★★	★★	★★★	7/9
[34]	Case-Control	★★★★	★★	★★★	9/9
[40]	Case-Control	★★	★	★★★	6/9
[36]	Case-Control	★★★★	★★	★★	8/9
[37]	Case-Control	★★★	★★	★★	7/9
[38]	Case-Control	★★★	★★	★★★	8/9
[39]	Case-Control	★★★★	★★	★★★	9/9
[40]	Case-Control	★★★★	★★	★★	7/9
[41]	Case-Control	★★	★★	★★★	7/9
[42]	Case-Control	★★★	★★	★★	7/9
[43]	Case-Control	★★★	★★	★★	7/9
[44]	Case-Control	★★	★★	★★★	9/9

Criteria chosen for quality assessment of case-control studies.

Selection	Aceptable
Is the case definition adequate?	The cases are obtained from medical records, diagnosed by the hospital team or independently validated.
Representativeness of the cases	Representative of the average number of breast cancer patients in the community (only studies targeting patients with primary breast cancer were included)
Selection of controls	The controls come from the same community.
Definition of controls	The participants declare that they have no history of disease.

Comparability	Aceptable
Cases and controls are compared according to the design or analysis included in the study.	Both cases and controls are matched, or the result has been adjusted for 2-3 of the selected confounders (1 star).<br><br>Both cases and controls match, or the result has been adjusted for 4 or more of the selected confounders (2 stars).<br><br>Selected confounders: age, ethnicity/race, ER status, PR status, HER2 status, histologic grade, stage, biological sample, country.

Exposure	Aceptable
Was the exposure determined?	MicroRNA analysis was performed using a validated method (eg, RT-qPCR, microarray…).
Was the same method used for cases and controls?	Yes
Unnecessary exclusion of participants	There is no unnecessary exclusion of participants.

Study	Source	Country	Ethnicity	QUADAS Score	Endogenous control	RNA extraction	Median age BC patients
[47]	Serum	Germany	Caucasian	8	miR-16	total RNA	65
[49]	Urine	Switzerland	Caucasian	8	miR-16	small RNA molecules	52
[24]	Serum	Mexico	Caucasian	7	18S RNA	small RNA molecules	52
[37]	Serum	Egypt	Caucasian	8	miR-16	total RNA	50
[18]	Serum	China	Asian	7	miR-16	total RNA	51
[19]	Serum	China	Asian	8	RNU38B	total RNA	54
